# Hepatoprotective Effects of a Novel Trihoney against Nonalcoholic Fatty Liver Disease: A Comparative Study with Atorvastatin

**DOI:** 10.1155/2020/4503253

**Published:** 2020-10-09

**Authors:** Hamad Abdulsalam Hamad Alfarisi, Muhammad Bin Ibrahim, Zenab B. Hamad Mohamed, Nuraniza Azahari, Asmah Hanim Bt. Hamdan, Che Anuar Che Mohamad

**Affiliations:** ^1^Department of Nutrition Sciences, Kulliyyah of Allied Health Sciences, International Islamic University Malaysia, Kuantan 25200, Pahang, Malaysia; ^2^Department of Pathology and Laboratory Medicine, Kulliyyah of Medicine, International Islamic University Malaysia, Kuantan.25200, Pahang, Malaysia; ^3^Department of Basic Medical Sciences, Kulliyyah of Pharmacy, International Islamic University Malaysia, Kuantan.25200, Pahang, Malaysia

## Abstract

Nonalcoholic fatty liver disease (NAFLD) is the most prevalent chronic liver disorder worldwide with no curative therapy. The aim of this study was to investigate the hepatoprotective effects of a novel Trihoney against biochemical and histological manifestations of NAFLD in hypercholesterolemic rabbits. *Methodology*. Forty-eight male New Zealand white (NZW) rabbits were grouped into normal diet (C), normal diet with 0.6 g/kg/day of Trihoney (C + H), 1% cholesterol diet (HCD), 1% cholesterol diet with 0.3 g/kg/day of Trihoney (HCD + H_1_), 1% cholesterol diet with 0.6 g/kg/day of Trihoney (HCD + H_2_), and 1% cholesterol diet with 2 mg/kg/day of atorvastatin (HCD + At.). Animals were sacrificed after 12 weeks of treatment. Serum lipids and liver function test (LFT) were measured prior to and at the endpoint of the experiment for total cholesterol (TC), low-density lipoprotein (LDL-c), alanine aminotransferase (ALT), aspartate aminotransferase (AST), alkaline phosphatase (ALP), gamma-glutamyl transferase (GGT), and total bilirubin (T. Bil.). Liver was processed for histopathology study. Liver homogenate was analysed for oxidative stress parameters: superoxide dismutase (SOD), glutathione peroxidase (GPx), and malondialdehyde (MDA). *Results*. Lipid analysis approved the induction of hypercholesterolemia. A significant elevation (*p* < 0.01) of serum AST and ALT levels showed by the HCD group was compared to C and C + H groups. Trihoney exhibited a significant reduction (*p* < 0.001) of AST and ALT compared to the HCD group. Likewise, AST and ALT reduced significantly in the HCD + At. group (*p* < 0.001). Trihoney supplementation induced significant (*p* < 0.05) enhancement of SOD and GPx activities. Atorvastatin treatment was associated with significant (*p* < 0.05) reduction of SOD and GPx activities in the liver. Trihoney and atorvastatin showed marked (*p* < 0.001) reduction of hepatic lipid peroxidation. Trihoney showed histological protection against progression of NAFLD to nonalcoholic steatohepatitis (NASH). Atorvastatin exhibited no beneficial impact on hepatic architecture. *Conclusion*. Trihoney was able to maintain normal liver function and showed hepatoprotection against progression of NAFLD to NASH probably through hypocholesterolaemic and antioxidant functions.

## 1. Introduction

Nonalcoholic fatty liver disease is the most prevalent chronic liver disorder worldwide with no curative therapy [1]. Nonalcoholic fatty liver disease constitutes a spectrum of histological changes in the liver (due to fatty infiltration) ranging from simple steatosis to NASH (aggressive form) [2]. Differentiation between NAFLD and NASH can only be made on histological basis [3]. The global prevalence of NAFLD is 25.24% with highest prevalence in the Middle East and South America and lowest in Africa [4]. It is estimated that the prevalence of diagnosed cases of NASH will approach 18 million by 2027 in the USA, Japan, England, France, Germany, Italy, and Spain [1]. In Asia, the population prevalence of NAFLD is 25%, which is almost like that of Western countries, and this trend was attributed to the modern sedentary lifestyle and dietary habits [5]. The recently accepted theory explaining pathogenesis of NAFLD is the “multiple hit” theory which proposed multiple insults act together in genetically predisposed individual to induce NAFLD, and the multiple hits include insulin resistance, hormones secreted from the adipose tissue, nutritional factors, gut microbiota, and genetic and epigenetic factors [6]. Oxidative stress, lipid peroxidation, and inflammation are the underlying operating mechanisms for initiation and progression of NAFLD [6–9]. Despite its worldwide prevalence and clinical burden, to date, NAFLD has no curative pharmacological treatment [10]. Healthy lifestyle with correct dietary habits definitely maintains healthy status and protects against NAFLD [11]. Honey has been used as food and medicine by ancient and modern world and by all traditions and civilisations [12]. Natural honey has been reported to possess hepatoprotective properties through its unique constituents [13], antioxidant [14], and anti-inflammatory [15] functions. The aim of this study was to investigate the hepatoprotective effect of a novel Trihoney against biochemical and histological manifestations of NAFLD in hypercholesterolemic rabbits. Trihoney is a combination of Trigona, mellifera, and dorsata honey, the combination ratio was 45, 15, and 10 of the mentioned honey, respectively, and it was determined by Design Expert Version 6.0 software and response surface methodology (RSM) looking for a combination formula having the maximum total phenolic content (TPC) [16]. Trigona and dorsata honey are local Malaysian honey with proven high antioxidant capacities. Trigona has a sour taste because of that people may not tolerate it. However, it has a very high phenolic content and hence its powerful antioxidant function [17]. Dorsata honey is also proven to have a profound antioxidant function [18]. Because oxidative stress is documented as an underlying pathogenic mechanism in many diseases such as NAFLD/NASH, the aim was to formulate a combination from those potent antioxidant honey and making them tastily tolerable by adding to them mellifera honey. The TPC of Trihoney [(0.307 ± 0.004) mg GAE/g Trihoney] was superior on that of each individual honey. Moreover, Trihoney had high concentrations of phenolic compounds such as quercetin, kaempferol, rutin, catechin, maleic acid, caffeic acid, cinnamic acid, coumaric acid, gallic acid, *p*-hydroxybenzoic acid, salicylic acid, sinapic acid, and vanillic acid, in addition to high antioxidant properties such as ferric reducing ability of plasma analysis (FRAP) and DPPH free radical scavenging activity analysis [16]. The current study represents the first *in vivo* application of this combination which is suggested to provide synergistic effects with regard to the protective functions against NAFLD and NASH through antioxidant [19] and anti-inflammatory effects [16].

## 2. Materials and Methods

### 2.1. Chemicals and Reagents

Pure cholesterol powder is obtained from Nacalai-Tesque (Kyoto, Japan). Cholesterol-free extra virgin coconut oil is bought from Philippines. Masson's Trichrome (MT) stain kit is from Clin-Tech (UK). Other solvents, chemicals, and haematoxylin and eosin (H&E) stain were supplied by Sigma-Aldrich (USA) and Leica Biosystems (Germany).

### 2.2. Trihoney and Atorvastatin

Trihoney is a product made in Department of Nutrition Sciences laboratories of Kulliyyah of Allied Health Sciences, International Islamic University Malaysia (IIUM). Trihoney is a combination of three types of natural honey: Trigona, mellifera, and dorsata, at a ratio of 45%, 15%, and 10%, respectively [16]. Trihoney was administered to the respective animal groups by oral route. Two doses have been used (0.3 g/kg/day and 0.6 g/kg/day). These rabbit equivalent doses have been calculated based on the human daily recommended dose of honey which ranges from 0.1 to 0.2 g/kg [20, 21]. Using Reagan-Shaw et al. [22] principle, animal equivalent dose is calculated according to the following equation: (human equivalent dose × human K_*m*_ factor = animal equivalent dose × animal K_*m*_ factor), with the adult human K_*m*_ factor being 37 and the rabbit K_*m*_ factor being 12. K_*m*_ factor is a constant calculated based on normalisation of body surface area for accurate translation of drug doses between mammalian species [22]. Atorvastatin 40 mg film-coated tablets (Prague-Czech) were crushed into fine powder, reconstituted in 1 mL of distilled water, and given by oral gavage using clean syringe [23–25], at a dose of 2 mg/kg body weight [23, 26].

### 2.3. Preparation of 1% Cholesterol Diet

Preparation of 1% cholesterol diet was performed according to Alfarisi et al. [19] as follows: 40 g of pure cholesterol powder (Nacalai-Tesque, Kyoto, Japan) was emulsified in 80 mL (=80 g) of cholesterol-free extra virgin coconut oil (product of Philippines). The cholesterol emulsion evenly poured over 3,880 g of standard rabbit pellets (Perternakan Hong Lee Sdn. Bhd, Malaysia). The prepared food (1% cholesterol and 2% coconut oil rabbit pellet) was packed in zipped bags and kept at temperature of 20–22°C for use.

### 2.4. Animal

Forty-eight NZW rabbits of male gender were purchased from certified experimental animal supplier (A Sapphire Enterprise, Seri Kembangan, Selangor, Malaysia). Animal weight ranged from 2 to 2.5 kg, and animals' age was 20 weeks. Animals were randomly housed in stainless-steel cages designed for rabbits as a single rabbit per cage with free access to water and standard rabbits' pellet, in addition to the standard animal care housing condition of 12 hours dark/light cycle, temperature 15–21, and humidity 45–65%. The procedure of animal handling was conducted in accordance with the guidelines of Malaysian Code of Practice for the Care and Use of Animals for Scientific Purposes (AEPC) [27], and the protocol of this experiment was approved by the Institutional Animal Care and Use Committee of International Islamic University Malaysia (IACUC-IIUM) with ID approval (IIUM/IACUC-Approval/2017 (19)).

### 2.5. Experimental Study

Forty-eight male NZW rabbits were grouped into the following 6 groups: normal diet (C), normal diet with Trihoney dose of 0.6 g/kg/day (C + H), 1% cholesterol diet (HCD), 1% cholesterol diet with 0.3 g/kg/day of Trihoney (HCD + H_1_), 1% cholesterol diet with 0.6 g/kg/day of Trihoney (HCD + H_2_), and 1% cholesterol diet with 2 mg/kg/day of atorvastatin (HCD + At.). At the end of 12 weeks, animals were sacrificed at the animal surgical laboratory of central research and animal facility, International Islamic University Malaysia (CREAM)-IIUM. Induction of general anaesthesia was done by intramuscular injection of a combination of ketamine and xylazine [28] at doses of 50 mg/kg and 10 mg/kg, respectively [29]. Laparotomy and sternotomy were performed for full exposure of liver [30], and then through left ventricular approach, systemic perfusion was secured by infusion of ice-cold normal saline and euthanasia was achieved by exsanguination of blood through opened right atrium [31, 32]. Portal vein was cannulated for further hepatic perfusion with ice-cold 0.9% NaCl for achievement of full hepatic tissue clearance [33]. Liver was then released, immediately weighed, and reexamined in a container filled with iced-cold phosphate buffer saline (PBS). Liver tissue was cut into 3 pieces of 6 g each for later homogenate study [33] and immediately stored at −80°C (Haier Ult Freezer, China) until homogenisation [34]. Piece of right lobe from each animal was cut to ensure optimisation and consistency [35] and then immediately fixed in 10% neutral buffer formalin (NBF) [36, 37] for histopathology study.

### 2.6. Blood Samples and Serum Preparation

Blood was collected from the animals twice, one time on day zero for baseline investigation and for verification of any physiological differences between animals and the second time at the endpoint of the experiment [23, 28]. Blood was withdrawn from central ear artery [29], collected in plain tubes, allowed to clot at room temperature for 40 minutes [38], and then centrifuged (Centrifuge Universal 320R Hettich, Germany) at 4 by speed of 3500 rpm, for 15 minutes [39, 40]. Serum samples were immediately sent for biochemical analysis.

### 2.7. Liver Homogenate Preparation

Liver tissue was retrieved from −80°C freezer and defrosted. Using bullet blender homogeniser, liver tissue was homogenised to a ratio of 10% (w/v) [41] as follows: 100 mg tissue in 900 *μ*L PBS (Sigma-Aldrich, USA) [42] supplemented with protease cocktail inhibitor (Nacalai-Tesque, Japan) [43]. Homogenised tissue was centrifuged (ThermoFisher Scientific, Germany) for 15 minutes at 10,000 rpm at 4°C [42, 43]. The supernatant was stored immediately at −80°C for later assay [44]. Protein concentration in tissue homogenate was measured according to Coomassie (Bradford) protein assay method [43] using Coomassie Brilliant Blue (CBB) solution (Ready to Use) (Nacalai-Tesque, Japan).

### 2.8. Serum Biochemical Analysis

Serum samples for TC, ALT, AST, ALP, GGT, and T. Bil. were immediately analysed by automated analysis machine (Au480 Auto Analyser-Beckman Coulter, Inc.). Serum LDL-c was calculated according to Friedewald equation [45]: LDL*-c* *=* TC－HDL*-c*－(TG*/5)*mmol/L.

### 2.9. Antioxidant Study

#### 2.9.1. Lipid Peroxidation Assay in Liver Homogenate

Concentration of MDA in liver homogenate was determined quantitively using OxiSelect MDA Adduct competitive enzyme-linked immunosorbent assay (ELISA) according to the manufacturer's protocol (Cell Biolabs, USA).

#### 2.9.2. Antioxidant Enzyme Assay in Liver Homogenate

Superoxide dismutase enzyme activity was assayed using OxiSelect™ Superoxide Dismutase Activity Assay Kit (Cell Biolabs, USA). Enzyme activity as function of optical density (OD) was expressed as units/µL and calculated as percentage (%). Activity of GPx was assayed using GPx assay kit (Abnova, Taiwan). The principle of GPx assay was based on the decrease in NADPH (measured at 340 nm) which is proportionate to GPx activity. Activity of GPx in the homogenate was expressed in mU/mg tissue protein.

### 2.10. Histopathological Study Using H&E and MT Staining

Liver tissue was fixed in 10% NBF for at least 72 hours and then was processed in automated tissue processor (Leica Microsystem, Germany), clarified in xylene, and embedded by embedding Centre (Tissue-Tek® Tec™, Germany). Trimming and sectioning of 3 *μ*m thick ribbon were performed using a semiauto rotator microtome (Leica Microsystem, Germany) and mounted onto frosted glass microscope slides. Haematoxylin and eosin [46] and MT staining [47] were employed. Staging and grading of histopathological findings were performed according to Brunt et al. [48] grading and staging system of NASH.

### 2.11. Statistical Analysis

Statistical Package for Social Sciences (SPSS version 21 Chicago, Illinois, USA) software was used for data processing. Data were expressed as mean (*M*) and standard deviation (SD) and analysed by one-way analysis of variance (ANOVA). One-way ANOVA followed by the post hoc test was used for determination of any significant differences between means of two or more independent groups. Statistical significance was considered at *p* < 0.05. Correlations were investigated using Pearson's correlation coefficients (*r*).

## 3. Results

### 3.1. Effects of Trihoney and 1% Cholesterol Diet on Animal's Food Intake

**Figure 1:**
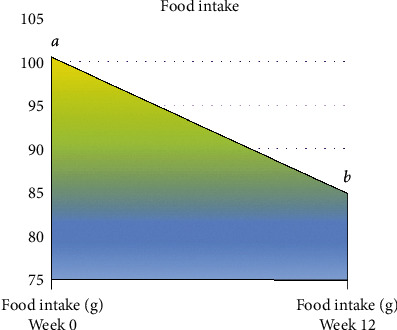
Follow-up of animal's food intake during the experiment. Values are means of 8 rabbits per group. The results of all experiment groups were analysed using repeated measure analysis of variance (repeated measure ANOVA), with multiple comparisons done using the Bonferroni test. Mean difference is considered significant at *p* < 0.05. ^a,b^Values differ significantly at *p* < 0.05.

Results of animal's food intake are displayed in Table 1. Continuous feeding and treating NZW rabbits with the designed regimen showed no significant (*p* > 0.05) difference between all experimental groups prior to induction phase (week 0) and after treatment phase (week 12). However, comparison by repeated measurements of animal's food intake pre- and posttreatment (Figure 1) showed a significant reduction (*p* < 0.05) in animals' food intake at the end of the treatment duration when compared to pretreatment starting point.

### 3.2. Trihoney Normalises Liver Function in NAFLD

Definitely, there was no difference (*p* > 0.05) in liver function parameters between all experimental groups before phase of induction (Table 2). While after 12 weeks of treatment (Table 2), the HCD group had a significant elevation (*p* < 0.001) of serum AST when compared to C and C + H groups and a significant elevation (*p* < 0.01) of serum ALT in comparison to the C group. More significant elevation (*p* < 0.001) of serum ALT level was seen between the HCD group and C + H group. Trihoney-treated groups showed significant reduction (*p* < 0.001) of both liver enzymes AST and ALT when compared to the HCD group. Likewise, the atorvastatin-treated group exhibited significant (*p* < 0.001) lower serum AST and ALT compared to the HCD group. Serum transaminases in the atherogenic-treated groups were comparable to the controls. Serum T. Bil. and GGT were lower in Trihoney-treated groups in comparison to HCD and HCD + At. groups despite no statistical significance.

### 3.3. Correlation between Liver Parameters and Serum Cholesterol

A significant (*p* < 0.05) positive correlation was found between serum AST, TC, and LDL-c levels. Moreover, T. Bil. had significant (*p* < 0.01) positive correlation with serum TC level and significant (*p* < 0.05) correlation with LDL-c (Table 3).

### 3.4. Trihoney Enhances Antioxidant Enzymes in NAFLD

There were a very significant (*p* < 0.001) reduction of SOD and GPx (*p* < 0.05) activities in the HCD group compared to the control group (Table 4). Contrarily, treatment group HCD + H_1_ exhibited the highest (*p* < 0.001) SOD activity among all atherogenic groups; moreover, this group showed the highest GPx activity despite no statistical significance difference compared to HCD and HCD + H_2_ groups. Activity of GPx was significantly (*p* < 0.05) enhanced in the HCD + H_1_ group compared to the HCD + At. group. Group HCD + H_2_ had SOD and GPx activities higher than the HCD and HCD + At. groups though no statistical significance difference. On the contrary, the atherogenic group received atorvastatin HCD + At. showed low SOD and GPx activities in liver homogenate when compared to all untreated and treated groups.

### 3.5. Trihoney Ameliorates Hepatic Lipid Peroxidation

High cholesterol diet induced significant (*p* < 0.001) elevation of MDA concentration in liver compared to the control groups (Table 4). Trihoney- and atorvastatin-treated groups showed significant (*p* < 0.001) reduction of MDA concentration in the liver compared to the HCD group. Both HCD + H_2_ and HCD + At. had liver MDA concentration comparable to that of the control groups.

### 3.6. Trihoney Exhibited Histological Protective Effects against Progression of NAFLD to NASH

#### 3.6.1. Macroscopic Examination

**Figure 2:**
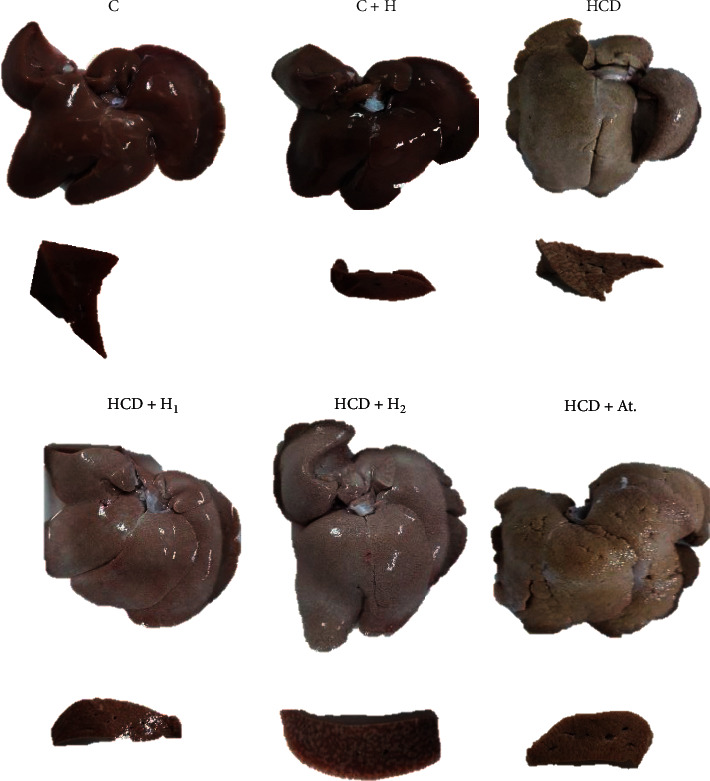
Macroscopic features and cut surface of a representative liver from each experimental group.

Macroscopically (Figure 2), control groups C and C + H showed similar gross morphological features comprised shiny deep maroon colour, smooth surface, soft consistency, and deep maroon cut surface. The liver of the HCD group had yellowish discolouration, smooth surface, hard consistency, and yellowish-brown cut surface. Trihoney-treated groups exhibited pale to yellow surface colour, soft consistency, and brown cut surface. Atorvastatin-treated group showed rough yellow-coloured surface, hard consistency, and yellowish-brown cut surface.

#### 3.6.2. Microscopic Study

**Figure 3:**
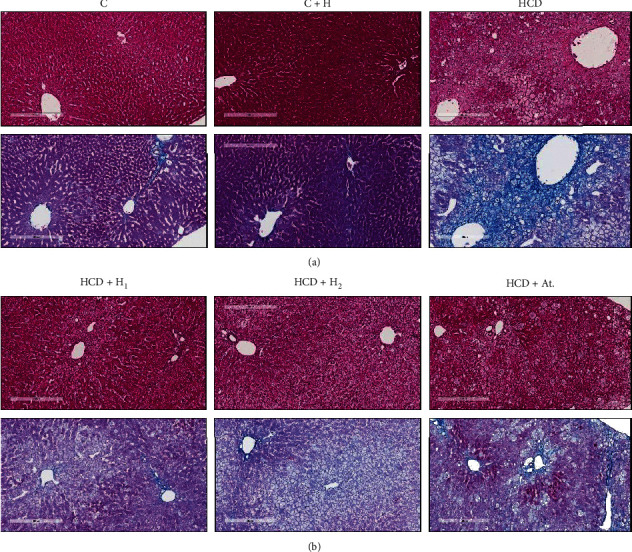
Microscopic features of a representative liver section from each experimental group: Haematoxylin and eosin stain (a); Masson's trichrome stain (b).

Microscopically (Figure 3), H&E staining of liver tissue of C and C + H groups showed normal hepatic tissue architecture. Staining by MT showed normal distribution of collagen fibres around the hepatic lobules and around the portal triads. Contrary, H&E staining of liver tissue of the HCD group demonstrated histological picture of NASH composed of macro- and microvesicular steatosis, ballooning, hepatic lobular inflammation with inflammatory infiltration by lymphocytes, plasma cells, occasionally neutrophils, and very prominent Mallory–Denk bodies. Masson's trichrome staining of this group showed fibrotic histopathological changes involved in acinar zones 1, 2, and 3, indicating advanced course of the disease, in addition to extensive hepatic fibrosis in the form of perisinusoidal, pericellular fibrosis giving picture of “chicken wire” which is typical of NASH. Moreover, MT staining highlighted deposition of collagen in periportal areas and showed bridging of fibrosis. Collectively, these histopathological findings are consistent with grade-2 (moderate) NASH and fibrotic stage-3. On the contrary, H&E staining of liver tissue of the Trihoney-treated groups showed evidence of NAFLD and focal mild grade-1 NASH (macro- and microvesicular steatosis, hepatic lobular inflammation, inflammatory cell infiltrates, no Mallory–Denk bodies, and no hepatocellular ballooning). Masson's trichrome staining of these groups showed histopathological changes involved in acinar zone 3 in the form of very minimal perisinusoidal fibrosis indicating early phase of the NAFLD fibrotic stage-1. Haematoxylin and eosin staining of HCD + At. liver tissue and MT staining showed histopathological grade-2 (moderate) NASH and fibrotic stage-3.

## 4. Discussion

Normal range of food intake in rabbits varies and is determined by factors such as animal's age, activity, and other environmental factors [49]. In this experiment, animals were allowed to feed on 160 g/day throughout the experimental period [49]. In this study, weekly food intake measurements showed gradual reduction in food intake by all groups of animals and throughout the experiment without any significant difference between them. Interestingly, gradual reduction of food intake when accompanied with normal growth and weight gain is considered as the normal physiological dietary and growth behaviour for NZW rabbits [50]. These results indicated that the status of food intake in this experiment is consistent and homogeneous among all animals, and the supplemented food will not alter the outcome of this study.

Elevated biochemical liver parameters are considered as a diagnostic tool in the setting of NAFLD [51]. Elevated serum levels of ALT, AST, and other parameters of LFT are used as indicators for hepatocellular damage [52]. Alanine aminotransferase is more specific and preferred over AST in the diagnosis of liver injury [53]. Alanine aminotransferase is more related to pathology of liver fat [54]. This model of hypercholesterolemic rabbits appeared to be an ideal model for the study of NAFLD because it showed biochemical as well as the histopathological derangement of NAFLD/NASH. This designed experiment is supported by Kainuma et al. [55] who reported that cholesterol-fed rabbits are useful models for the study of the pathophysiological aspects of NAFLD/NASH. In this model, the type of induced liver injury was a hepatocellular type. This is based on the results of the high cholesterol diet group that showed ALT serum level is > 2 of the upper limit of normal (ULN) [56]. The criteria for identification of drug-induced liver injury have been modified to include the following: ALT ≥3 ULN and total serum bilirubin ≥2 ULN [53], which were also applicable to the current model.

In this experiment, feeding of rabbits with 1% cholesterol diet for 12 weeks resulted in a very extensive elevation of hepatic ALT and AST in the high cholesterol diet group when compared to the control groups. This is consistent with investigation conducted by Kim et al. [57] who reported altered liver enzymes after feeding the NZW rabbits with 1% cholesterol for 3 months. But the results of the present study are not consistent with Kainuma et al. [55] who reported the transaminases had not been changed in the NAFLD model. Even though, the diagnostic role of liver AST and ALT in the setting of NAFLD is debatable [58], but up to date, they are still considered among the diagnostic biochemical markers of NAFLD [3].

This investigation demonstrated the protective role of Trihoney against impaired liver function in NAFLD/NASH complex. Treatment groups received Trihoney expressed normal serum AST and ALT and were very comparable to the control groups. Trihoney seems to reduce liver transaminases in concurrent hypercholesterolemia in a dose-dependent pattern.

Moreover, in this experiment, no significant difference was observed between all groups for serum ALP, GGT, and bilirubin, but treatment groups received Trihoney showed lower serum levels of these parameters in comparison to the high cholesterol diet group. Interestingly, the animal group which received Trihoney with normal diet expressed low serum levels of all hepatic biochemical markers (AST, ALT, GGT, ALP, and total bilirubin) in comparison to the control group that was maintained only on commercial diet. This may indicate the medical beneficial role of Trihoney even in normocholesterolemic status. These findings are supported by other animal studies investigating the hepatoprotective role of honey [14, 59, 60].

Statins are recommended treatment option for NAFLD as the dyslipidemia controlling agent because 70% of patients with NAFLD was found to have dyslipidemia [61]. Use of statins in the management of NAFLD is strictly limited to certain criteria determined by the histopathological picture of NAFLD/NASH [1]. In a randomised clinical trial study investigating combination of atorvastatin with vitamins C and E for 4-year duration in individuals with NAFLD, the results showed reduction in the risk of having moderate to severe steatosis by 70% [61]. This may indicate synergistic effect can be obtained if atorvastatin is combined with natural antioxidants. In our experiment, supplementation of 2 mg/kg/day atorvastatin along with 1% cholesterol diet showed significant reduction in serum AST and ALT levels. These findings are consistent with prospective studies conducted on NAFLD and NASH patients having dyslipidemia in order to evaluate atorvastatin as treatment choice for NAFLD and NASH, and the authors reported the efficacy of atorvastatin in normalising transaminases serum levels [62, 63]. In the current investigation, the atorvastatin-treated group expressed higher serum total bilirubin and GGT levels in comparison to other atherogenic groups even though elevation was statistically not significant but might indicate negative impact exerted by atorvastatin on NAFLD established disease. Elevated serum GGT is an indicator of hepatobiliary damage [64]. Safety and risk of statins in cases of liver diseases are still debatable. Atorvastatin reported in some cases to induce acute elevation of serum transaminases; however, it was transient and self-limiting in some cases and was severe hepatotoxicity in some others [65, 66]. In a large cohort 4-year follow-up study, conducted by Chang et al. [67] on patients with chronic liver disease and being on statin treatment, the authors reported that atorvastatin in large doses can increase the risk of hospitalisation due to sever liver injury; otherwise, statins are still safe. The latest recommendations from United States Federal Drug Administration (US FDA) stated that statins are associated with very low risk of serious hepatic injury [68].

In this study, we reported evidence of oxidative stress by investigating MDA concentration in homogenate of the liver. In line with our study, elevated MDA in NAFLD/NASH has been reported by various experimental as well as by clinical studies [69–74]. Pathophysiology of NAFLD is influenced by many environmental and genetic factors of which oxidative stress appeared as the main primary insult in starting and in progressing hepatic and extrahepatic damages [75]. In conjunction with high MDA concentrations, the HCD group had suppressed SOD and GPx activities in liver homogenate. In agreement with these findings, Xiao et al. [74] by in vitro and in vivo studies showed that, in status of NASH progression, the MDA production increased, while the protein expression of SOD and GPx suppressed. This is supported by Koruk et al. [76] who investigated oxidative stress in NASH patients, and the authors reported suppressed SOD along with elevated MDA in NASH subjects in comparison with healthy subjects. Interestingly, we reported significant results pertaining antioxidant potential of Trihoney. Supplementation of Trihoney resulted in a protective antioxidant effect expressed as enhancement of SOD and GPx activities in the liver despite presence of NAFLD/NASH. Concurrently, Trihoney supplementation exhibited significant ability to reduce lipid peroxidation and oxidative stress by suppression of MDA concentration in liver tissue. In agreement with our reported antioxidant-hepatoprotective results, Xiao et al. [74] showed that natural honey can alleviate hepatic oxidative stress by inhibition of MDA production and by reducing protein expression of both SOD and GPx enzymes. In line with our reports, natural honey was investigated by Kilicoglu et al. [15] for its role against obstructive jaundice-induced hepatic oxidative stress, and the authors concluded that natural honey expressed significant antioxidant function by suppression of hepatic MDA production in addition to histological protective effects. Natural honey exhibited antioxidant-hepatoprotective effects against aflatoxin-induced liver injury by restoration of MDA concentration and SOD activity to the normal range in the liver tissue which supports our results [60]. One of the underlying hepatoprotective mechanisms implicated in this setting is the inhibition of thioredoxin binding protein (TXNIP) overexpression. Thioredoxin binding protein is an important regulator for redox homeostasis, and reduction in its expression can suppress ROS production [77]. We may link the antioxidant to the anti-inflammatory effects of honey through TXNIP. Thioredoxin binding protein is found to be able to activate Nod-like receptor protein 3 (NLRP3) inflammasome which is implicated in inflammatory process and in progression of NASH. Xiao et al. [74] suggested that honey exerted its hepatoprotective effect by antioxidant and anti-inflammatory properties through inhibition of TXNIP, which subsequently suppresses NLRP3 inflammasome. The antioxidant potential of honey has been attributed in part to its polyphenolic contents [78]. Liu et al. [70] reported antioxidant effect of blue honeysuckle against NAFLD and attributed hepatoprotective effect to the blue honeysuckle polyphenolic contents. We may postulate that Trihoney has potential hepatoprotective effects against NAFLD/NASH through antioxidant function. Trihoney antioxidant effect may be attributed in part to its high phenolic contents for which it was formulated, in addition to other possible molecular mechanisms requiring future studies.

In this investigation, atherogenic group treated with atorvastatin expressed suppressed SOD and GPx activities in the liver coincidently with reduced lipid peroxidation manifested by low MDA concentration in hepatic tissue. Regarding hepatoprotective function of atorvastatin in this investigation, we found the following: reduced lipid peroxidation and normal biochemical liver function; these two effects may be consistent to indicate the role of atorvastatin in management of NAFLD, but simultaneously, we reported marked suppression of SOD and GPx activities in the liver in addition to the nonprotective histological effect; collectively, these results make the effects of atorvastatin in NAFLD inconsistent and nonconclusive and necessitate future research studies to further explain and resolve this issue. In line with this conclusion, Sigler et al. [79] showed evidence by a comprehensive review of a recent 12 clinical trials that statins are safe to be used in NAFLD patients, but their specific prescription for NAFLD treatment remained unclear due to controversy of liver histological outcomes. In accordance with our findings, Hyogo et al. [63] and Gómez-Domínguez et al. [80] reported that statins had significant reducing effect on MDA in addition to significant improvement in liver biochemical function, but the authors ignored antioxidant enzymes assay; additionally, they reported some patients had unexplained progression in liver fibrosis. In a randomised clinical trial investigated effect of combination of atorvastatin with vitamins C and E as antioxidants in NAFLD subjects, Foster et al. [61] showed significant reduction of liver steatosis in NAFLD subjects after 4 years of active therapy, but the study did not include antioxidant effect of atorvastatin. Such study encourages us to propose combination of Trihoney with atorvastatin as a therapeutic regimen for hyperlipidemic NAFLD patients. Such adjuvant therapy may protect against the unwanted histological side effects of atorvastatin, and the patients may get benefit of other pleiotropic functions of honey.

As far as histopathology of NAFLD is concerned, histopathological examination remained the cornerstone diagnostic tool for this disease [3]. In this study, we succeeded to induce NAFLD/NASH complex in NZW rabbits by feeding them 1% cholesterol diet for 12 weeks duration. Dyslipidemia and hypercholesterolemia are well-known leading causes to NAFLD [10, 81]. Our model of NAFLD may be considered as ideal for translational investigations because it showed the spectrum from steatosis to the fibrosis. The reported macroscopic examination of the liver in the current study showed difference between various groups, and the difference could be attributed to the impact of high cholesterol diet on the pathological process induced in the liver. Both HCD and HCD + At. groups showed similar gross findings. Our mentioned gross findings were in agreement with Xiaomin Wang et al. [82] who investigated NAFLD-induced high cholesterol diet. These gross findings were very consistent with the microscopic picture demonstrated by H&E and MT staining of liver tissues. These experimental groups had the full spectrum of NAFLD/NASH (steatosis, ballooning, inflammation, and hepatic fibrosis) in addition to presence of Mallory–Denk bodies in abundance, indicating the severity of NASH. According to Brunt et al. [48] grading and staging system of NASH, these experimental groups fall in grade-2 and fibrotic stage-3. The positive correlation between Mallory–Denk bodies and severity of steatosis and hepatitis was reported by Kayaçetin [83], which supports our histopathological description. In this study, use of atorvastatin showed no protective effect on histological picture of NAFLD in hypercholesterolemic rabbits, but serum transaminases maintained within normal and serum lipids (lipid profile not shown) significantly reduced; this is in line with Hyogo et al. [63], who reported normalisation of serum transaminases, while the histological picture of NASH improved in some patients and got worse in some others due to progression of fibrosis. Gómez-Domínguez et al. [80] reported the effectiveness of atorvastatin in lowering liver transaminases, but no effect is shown on liver fat contents, which support our results. Recently, Cioboată et al. [62] showed effectiveness of atorvastatin in normalising liver transaminases, but no impact was reported on hepatic fibrosis although they reported some reduction in steatosis. Our findings will add to the debate about use of statins in NASH patients. The debate and contradiction among experimental and clinical trials about the efficacy of hepatoprotective and therapeutic role of statins for NASH is still existing [1]. In clinical practice, it a very common to come across patients having hypercholesterolemia and cardiovascular diseases with concurrent NAFLD and NASH. In such cases, clinical practitioners have to decide about the use of statins, which represent an integral part of the regimen of such patients. In the current study, we used atorvastatin for this purpose and to add a knowledge about the safety of statins in status of hypercholesterolemic subjects having NAFLD or NASH. This aim was achieved by the current study, and it was supported by the literature. The current study indicated that, in subjects having hypercholesterolemia with NAFLD or NASH, the liver function should be monitored, and hepatic histology should be followed up for possibility of worsening of fibrosis.

On the contrary, macroscopic features of the liver in Trihoney-supplemented groups were in pale yellowish colour and had smooth surface, sharp edge, brown cut surface, and smooth consistency. These gross findings were in line with the microscopic histological features of these tissues. These groups had (mild) grade-1 NASH and fibrotic stage-1 according to Brunt et al. [48] grading and staging system of NASH. In this study, we may assume that Trihoney had potential hepatoprotective effect against progression of NAFLD to NASH. To the best of our knowledge, this is the first study that used this novel Trihoney for testing its hepatoprotective potential in hypercholesterolemia-induced NAFLD. As far as hepatoprotective effect of honey is concerned, Erejuwa et al. [14] reported the hepatoprotective effect of Malaysian Tualang (dorsata) honey in a diabetic rat model, but the study was biochemical and not histopathological. Korkmaz and Kolankaya [84] used the rat model and mentioned the histological protective effect of Anzer honey (Turkish honey) against liver injury induced by *N*-ethylmaleimide. Hepatoprotective histological effect of natural honey was reported also by Yaman et al. [60] who investigated honey versus aflatoxin in the rat model. The authors of those studies attributed the hepatoprotective effect of honey to its antioxidant properties. In the rat model with induced NAFLD, honey was investigated for its ability to ameliorate the histopathological changes in NASH, and the authors attributed the hepatoprotective effects of honey to its antioxidant and anti-inflammatory properties [74]. This is very consistent with our investigations. In a study on the rabbit model of NAFLD, the authors investigated flavonoid from grape and they reported a promising effect in minimising histological signs of NAFLD [85], and this supports our study in terms of role of natural flavonoids in protection against NAFLD. In a study using mice NAFLD model, the authors investigated the protective effect of blue honeysuckle extract against NAFLD; they characterised that extract as rich in polyphenols and showed hepatoprotective results against NAFLD, explored the antioxidant mechanism, and assigned it as the most likely underlying protective mechanism [70]. Likewise, Trihoney is a combination rich in phenolic compounds [16], and we may assume that the hepatoprotective effect of Trihoney may attribute to its antioxidant function. The exact molecular mechanisms need further studies. From our study, we can integrate both biochemical and histological effects of Trihoney as protective effects, but atorvastatin showed biochemical protective effect and no structural protective impact on liver tissue in NAFLD/NASH.

Since inflammation and oxidative stress secondary to hepatic lipid accumulation are implicated in the pathogenesis of NAFLD/NASH [6], and because the hepatoprotective functions of honey could be attributed to the antioxidant [14, 59] and to the anti-inflammatory [86] properties of honey, Trihoney could normalise the liver function and protect against progression of NAFLD to NASH through antioxidant and anti-inflammatory effects. Trihoney through the antioxidant study on liver homogenate in this work was proven to possess a tremendous antioxidant function through enhancement of hepatic antioxidant enzymes such as SOD and GPx. Moreover, Trihoney showed a very potent antioxidant effect against lipid peroxidation. Furthermore, Trihoney reported to have a systemic antioxidant effect and anti-inflammatory function against some inflammatory mediators implicated in the pathogenesis of NAFLD and NASH [16, 19]. Concurrently, this experiment provided evidence of a strong positive correlation between hypercholesterolemia and transaminitis (elevated AST). We reported a lipid lowering effect of Trihoney in a previous work [87]; taking this in consideration, Trihoney may exert its hepatoprotective effect through hypocholesterolaemic, antioxidant, and anti-inflammatory mechanisms.

## 5. Conclusion

In a status of sustained hypercholesterolemia, Trihoney was an effective hepatoprotective supplement protecting against functional and structural changes in NAFLD. Trihoney was able to maintain liver biochemical parameters within the normal range and showed ameliorative potential against hepatic oxidative stress. Trihoney exhibited protective function against histological progression of NAFLD to NASH and restrains hepatic fibrosis. Trihoney may be introduced as an adjuvant therapy with atorvastatin for hypercholesterolemic NAFLD patients. Although this study showed the hepatoprotective functions of Trihoney against NAFLD, some underlying mechanisms at molecular and at immunohistopathological levels have not been addressed, which represents a limitation in this study. Future studies covering alpha-smooth muscle staining in addition to therapeutic experimental model assessment for Trihoney are highly recommended.

## Figures and Tables

**Table 1:** Effect of 1% cholesterol diet and Trihoney on animal's food intake.

Groups	Food intake (g), week 0	Food intake (g), week 12
C	112.86 ± 15.69^a^	106.86 ± 39.04^a^
C + H	118.38 ± 33.34^a^	89.50 ± 55.68^a^
HCD	100.87 ± 37.21^a^	74.50 ± 30.92^a^
HCD + H_1_	81.87 ± 35.93^a^	80.00 ± 42.50^a^
HCD + H_2_	99.50 ± 45.11^a^	80.25 ± 31.59^a^
HCD + At	85.25 ± 36.73^a^	80.28 ± 26.69^a^

Values are mean ± standard deviation (SD) of the mean. The results of all experiment groups were analysed using one-way analysis of variance (ANOVA), followed by the Tukey HSD post hoc test. Mean difference is considered significant at *p* < 0.05. ^a^Values not sharing a common superscript letter within the same column differ significantly at *p* < 0.05. C = control and C + H = control group received a honey dose of 0.6 g/kg. HCD = high cholesterol diet; HCD + H_1_ and HCD + H_2_ = atherogenic groups received a honey dose of 0.3 and 0.6 g/kg, respectively; HCD + At = atherogenic group received atorvastatin dose of 2 mg/kg.

**Table 2:** Baseline (week 0) and final (week 12) serum liver function test.

Parameters	Groups											
	C		C + H		HCD		HCD + H_1_		HCD + H_2_		HCD + At.	
	Week 0	Week 12	Week 0	Week 12	Week 0	Week 12	Week 0	Week 12	Week 0	Week 12	Week 0	Week 12
Total Bil. (*µ*mol/L)	2.33 ± 1.37	4.00 ± 2.09	3.17 ± 2.79	3.17 ± 1.47	3.83 ± 1.94	16.17 ± 5.23	3.00 ± 3.16	4.67 ± 2.73	3.67 ± 2.66	12.67 ± 7.23	3.17 ± 2.71	25.33 ± 29.69
ALP (U/L)	160.50 ± 126.48	93.67 ± 30.22	95.33 ± 35.43	73 ± 37.87	77.17 ± 29.34	73.83 ± 37.49	142.83 ± 78.85	49.67 ± 29.04	134.17 ± 34.59	55.50 ± 13.09	98.50 ± 23.17	52.17 ± 26.33
GGT (U/L)	10.67 ± 3.56	12.17 ± 0.98	10.00 ± 2.00	10.83 ± 6.15	9.00 ± 2.45	14.83 ± 3.31	10.67 ± 3.98	5.83 ± 2.14^d^^*∗*^	12.17 ± 4.49	13.50 ± 8.41	10.50 ± 2.95	24.17 ± 22.39
AST (U/L)	48.67 ± 18.75	55.67 ± 21.23	50.67 ± 12.61	51.17 ± 24.74	49.50 ± 15.86	200.00 ± 64.56^a, b^*∗∗∗*	62.33 ± 22.43	62.33 ± 48.09^c^^*∗∗∗*^	52.00 ± 12.44	48.83 ± 35.39^c^^*∗∗∗*^	39.33 ± 11.74	56.17 ± 47.66^c^*∗∗∗*
ALT (U/L)	66.17 ± 22.78	71.50 ± 36.69	53.50 ± 19.85	41.83 ± 8.30	60.67 ± 7.45	180.17 ± 59.25^*a∗∗*^^, b^*∗∗∗*	59.83 ± 17.01	56.83 ± 50.19^c^^*∗∗∗*^	64.83 ± 14.82	48.83 ± 26.51^c^^*∗∗∗*^	43.83 ± 14.99	55.50 ± 36.31^c^*∗∗∗*

Values are mean ± standard deviation (SD) of the mean. The results of all experiment groups were analysed using one-way analysis of variance (ANOVA), followed by the Tukey HSD post hoc test. Mean difference is considered significant at *p* < 0.05.ALT, alanine aminotransferase; AST, aspartate aminotransferase; ALP, alkaline phosphatase; GGT, gamma-glutamyl transferase; T. Bil., total bilirubin. ^a^Significant different from the C group; ^b^significant different from the C + H group; ^c^significant different from the HCD group; ^d^significant different from HCD + At. *∗p* < 0.05; *∗∗p* < 0.01; *∗∗∗p* < 0.001].

**Table 3:** Correlation (*r*) between serum liver parameters and lipid profile at week 12.

	TC (mmol/L)	LDL-c (mmol/L)
AST (U/L)	*r* = 0.350^∗^	*r* = 0.398^*∗*^
T. Bil. (*µ*mol/L)	*r* = 0.437^∗∗^	*r* = 0.410^*∗*^

Values are the Pearson correlation coefficient (*r*) between serum level of liver biochemical parameters and lipid profile at week 12. *r* is the Pearson correlation coefficient. ^∗^*p* < 0.05; ^∗∗^*p* < 0.01 Sig. (2-tailed).

**Table 4:** Effect of Trihoney on activities of SOD and GPx and on MDA concentration in liver homogenate of NAFLD at week 12.

Group	SOD activity (%)	GPx activity (mU/mg protein)	MDA (*µ*g/mg protein)
C	61.43 ± 2.69	109.88 ± 40.44	0.00 ± 0.00
C + H	46.89 ± 2.44	90.97 ± 37.26	0.03 ± 0.01
HCD	27.98 ± 7.05^a,b∗∗∗^	49.98 ± 36.40^a∗^	0.98 ± 0.17^a,b∗∗∗^
HCD + H_1_	50.95 ± 5.15^a∗^^,c∗∗∗^	95.68 ± 13.77	0.31 ± 0.12^a,b∗∗^^,c∗∗∗^
HCD + H_2_	29.89 ± 10.62^a∗∗∗^^,b∗∗^^,d∗∗∗^	72.45 ± 22.16	0.14 ± 0.04^c∗∗∗^^,d∗^
HCD + At.	26.43 ± 5.49^a,b,d∗∗∗^	37.45 ± 33.66^a^^,b∗^^,d∗^	0.08 ± 0.13^c∗∗∗^^,d∗∗^

Values are mean ± standard deviation (SD) of the mean. The results of all experiment groups were analysed using one-way analysis of variance (ANOVA), followed by LSD post hoc test. Mean difference is considered significant at *p* < 0.05. ^a^Significant different from the C group; ^b^significant different from the C + H group; ^c^significant different from the HCD group; ^d^significant different from the HCD + H_1_ group. ^∗^*p* < 0.05; ^∗∗^*p* < 0.01; ^∗∗∗^*p* < 0.001.

## Data Availability

The data used to support the findings of this study are included within the article.
